# Facilitators and Barriers of Antiretroviral Therapy Initiation among HIV Discordant Couples in Kenya: Qualitative Insights from a Pre-Exposure Prophylaxis Implementation Study

**DOI:** 10.1371/journal.pone.0168057

**Published:** 2016-12-08

**Authors:** Rena C. Patel, Josephine Odoyo, Keerthana Anand, Gaelen Stanford-Moore, Imeldah Wakhungu, Elizabeth A. Bukusi, Jared M. Baeten, Joelle M. Brown

**Affiliations:** 1 Division of Allergy and Infectious Diseases, Department of Medicine, University of Washington, Seattle, WA, United States of America; 2 Centre for Microbiologic Research, Kenya Medical Research Institute, Kisumu, Kenya; 3 Northwestern University, Chicago, IL, United States of America; 4 School of Medicine, University of California, San Francisco, CA, United States of America; 5 Departments of Obstetrics and Gynecology and Global Health, University of Washington, Seattle, WA, United States of America; 6 Departments of Epidemiology, Global Health, and Medicine, University of Washington, Seattle, WA, United States of America; 7 Departments of Epidemiology and Biostatistics and Obstetrics, Gynecology, and Reproductive Sciences, University of California, San Francisco, CA, United States of America; London School of Hygiene and Tropical Medicine, UNITED KINGDOM

## Abstract

**Introduction:**

The World Health Organization now recommends antiretroviral therapy (ART) initiation for all HIV-infected individuals regardless of CD4 cell count or disease status. Understanding the facilitators and barriers to initiation of and adherence to ART is essential to successful scale-up of “universal” ART.

**Methods:**

To investigate facilitators and barriers to ART initiation, we conducted 44 in-depth individual or couple interviews with 63 participants (33 participants with HIV and 30 without HIV) already enrolled in a prospective implementation study of oral antiretroviral-based prevention in Kisumu, Kenya between August and September 2014. A semi-structured interview guided discussions on: 1) perceived advantages and disadvantages of ART; 2) reasons for accepting or declining ART initiation; and 3) influence of prevention of transmission to partner or infant influencing ART use. Transcripts from the interviews were iteratively analyzed using inductive content analysis.

**Results:**

HIV-infected participants indicated that living a healthier life, preventing HIV transmission to others, and appearing “normal” or “healthy” again facilitated their initiation of ART. While appearing “normal” allowed these individuals to interact with their communities without stigmatization, they also perceived community opposition to their initiating ART, because appearing “normal” again prevented community members from easily identifying infected individuals in their community. Denial of diagnosis, disclosure stigma, perceived side-effects, and challenges in obtaining refills were additional barriers to ART initiation.

**Conclusions:**

Community perceptions play an important role in both facilitating and inhibiting ART initiation. Perceived stigma, including perceived community opposition to widespread ART use, is an important barrier to ART initiation. Addressing such barriers, while capitalizing on facilitators, to ART initiation should be central to universal ART scale-up efforts.

## Introduction

HIV serodiscordant couples are an important target population for HIV prevention. Serodiscordant couples constitute nearly 50% of the couples in East Africa where at least one individual is HIV-infected, and they account for a substantial number of new HIV infections in this region [1–3]. The use of anti-retroviral treatment (ART) is not only associated with decreased HIV-related morbidity and mortality and increased quality of life for those infected with HIV [4, 5], but appropriate ART use significantly reduces the risk of HIV transmission to the uninfected partner [6, 7]. Agreeing to initiate and adhere to ART, however, is paramount to ART effectiveness [8–10].

The World Health Organization (WHO) now recommends ART initiation for all HIV-infected individuals regardless of CD4 cell count or disease status [11]. Understanding the facilitators and barriers to initiation of and adherence to ART is essential to successful scale-up of “universal” ART. In studies so far, several system-level barriers to ART use, such as requirement for CD4 testing, delay in enrolling in medical care facilities after testing, or lack of sufficient quantities of drugs, have been identified [9, 12, 13]. With the current recommendations for and programmatic scale-up of universal ART, however, studies focused on individual level barriers from resource-limited settings are urgently needed.

We conducted a qualitative study among heterosexual discordant couples enrolled in a prospective implementation study of oral antiretroviral-based prevention in Kisumu, Kenya. At the time of this qualitative study, 20% of those who were HIV-infected and qualified for ART initiation had not initiated ART. Thus, the objectives of our study were to identify facilitators of and barriers to initiation of and anticipated adherence to ART among infected individuals in discordant relationships in a resource-limited setting.

## Materials and Methods

The study was conducted in Kisumu, Kenya between August and September 2014. Kisumu County has one of the highest HIV prevalence estimates in Kenya at 19.3%, compared with 6.0% nationally [14]. Participants in this qualitative study were already enrolled in the Partners Demonstration Project in Kisumu. The Partners Demonstration Project is an open-label study of antiretroviral-based HIV prevention implemented at four sites in Kenya and Uganda among 1013 high risk HIV discordant couples [15]. HIV serodiscordant couples with high HIV transmission risk were enrolled; HIV-infected partners could not be using ART at enrollment to be eligible for the study. After enrollment, ART-eligible HIV-infected partners were referred to local HIV facilities to initiate ART per country guidelines, while the uninfected partner was offered pre-exposure prophylaxis (PrEP) as a “bridge” until the infected partner became eligible for and took ART for six months.

For this qualitative study, we selected a quasi-random subsample of the participants enrolled in the Partners Demonstration Project in Kisumu who fell into one of four categories: 1) HIV-infected individual eligible for ART who initiated ART; 2) HIV-infected individual eligible for ART who declined ART initiation; 3) HIV-uninfected individual eligible for PrEP who initiated PrEP; and 4) HIV-uninfected individual eligible for PrEP who declined PrEP initiation. In this paper, we present findings on facilitators and barriers to ART initiation reported largely by the HIV-infected individuals who initiated or declined to initiate ART. Other findings, such as facilitators and barriers to PrEP initiation, are presented elsewhere [16]. We assessed initiation of ART or PrEP by the third month of the study, anticipating that this was sufficient time for individuals to undergo counseling and decide on initiation of ART or PrEP. At the time of determining eligibility for this qualitative study in June 2014, ART eligibility was recommended for individuals with CD4 cell counts ≤350 cells/uL or >350 cells/uL with a WHO clinical disease stage III or IV [17]. After generating lists of potential participants in each of the four above categories, we randomly selected 20 participants to sample for this qualitative study with the goal of conducting at least 10 interviews in each category. From this random sample, we attempted to invite an equal number of male and female participants, however, some of the categories were highly skewed by gender, e.g. individuals who initiated ART were largely female. These potential participants were contacted by phone and, if interested, scheduled to return to the study facility with their study partner for an interview.

By the end of June 2014, a total of 251 couples were enrolled in the Partners Demonstration Project in Kisumu. Out of the 80 individuals invited to participate in this qualitative study, we conducted 44 in-depth interviews with a total of 63 participants (30 participants without HIV and 33 with HIV). Nineteen of the interviews were conducted with the couples together and an additional four interviews were conducted with couples but individually; the remainder 21 interviews were conducted with only one individual from a couple. The interviewers were trained to specifically elicit comments from both partners and reflections on each others’ responses during couple interviews.

The interviews were conducted by two trained interviewers, one female and one male, in DhoLuo and digitally recorded. We developed a semi-structure interview guide roughly informed by the Health Belief Model (particularly “perceived benefits” and “perceived barriers”) [18] and the Theory of Planned Behavior (particularly “behavioral intention” and "subjective norms”) [19]. The interviewers used this guide to prompt discussions on the following themes: 1) perceptions of ART, including advantages and disadvantages of each; 2) reasons for accepting or declining ART initiation; and 3) influence of prevention of transmission to partner or infant influencing ART/PrEP use. The interviewers then transcribed the initial five interviews in DhoLuo and then translated these into English. Another member of the study staff verified the accuracy of the English translations against the audio file and DhoLuo transcripts. Thereafter, the interviewers translated the interview audio files directly into English. Transcripts were imported into Nvivo 10.1 for coding [20]. Transcripts from the interviews were iteratively analyzed using inductive content analysis. An initial codebook was developed from the interview guide, which was further refined with discussion and consensus as the initial transcripts were coded. The first ten transcripts were double coded by at least two members of the study team and differences in coding were resolved through discussion until consensus was reached. After all the data were coded, the investigators used an iterative process of reading transcripts, applying inductive codes, comparing and contrasting codes, and identifying convergent and divergent themes.

The study received approval from the Kenya Medical Research Institute and University of California, San Francisco ethics review boards. All participants provided written informed consent.

## Results

Of the 33 HIV-infected participants interviewed, all were eligible for ART, 23 (70%) initiated ART during the study, 24 (73%) were female, 31 (94%) were married, and 31 (94%) were of Luo ethnicity. The median age was 32 years (IQR 23–37), the median total number of living children was of 1 (1–3) and number of living children with their study partner was 0 (0–2). The participants had been cohabitating with their study partner for a median number of 2 years (1.0–6.3) and had known their discordant status for a median of 1 month (1–1; see Table 1).

**Table 1:** Baseline characteristics of participants.

Variable	HIV-infected and eligible for ART(n = 33)
Age (years)	32 (23–37)
Gender	
Female	24 (73%)
Male	9 (27%)
Married	31 (94%)
Number of living children	1 (1–3)
Number of living children with study partner	0 (0–2)
Ethnicity	
Luo	31 (94%)
Luhya	2 (6%)
Kalenjin	0
Years of schooling completed	8 (7–12)
Monthly income for participant	20 USD* (0–49)
Number of years cohabitating with study partner	2 (1–6.3)
Number of months HIV-discordant status known	1 (1–1, range 1–13)
Number of months since first positive HIV test	9.5 (6.4–12.8)
Initiated ART during study	23 (70%)
Study partner on PrEP	22 (67%)
CD4 cell count (cells/μL)	305 (233–431)
Viral load (copies/mL)	71,174 (23,728–245,278)
WHO stage	
Stage 1	24 (73%)
Stage 2	9 (27%)
Stage 3	0
Stage 4	0

N (%) for categorical variables; Median (IQR) for continuous variables

*Conversion rate of 1KSh = 0.0098 USD used

Several major overlapping themes emerged from the interviews: 1) factors facilitating initiation of ART; and 2) barriers, both experienced or anticipated, that participants identified in initiating or adhering to ART (Table 2). Below we explore each theme in more detail.

**Table 2:** Facilitators and barriers to initiating ART among serodiscordant couples.

	Theme categories	Themes
**Facilitators**	*Individual and family benefits*	■ Initiating ART facilities living a longer and healthier life
		■ Use of ART prevents HIV transmission to partners or infants, and helps continue their relationships
	*Stigma-related*	■ Allows infected individuals to appear normal or healthy in order to avoid disclosure, stigma, and discrimination
**Barriers to initiating ART**	*Stigma-related*	■ Perceived opposition from the community or religious groups
		■ Denial about HIV positive diagnosis
		■ Stigma and fear of disclosure of HIV status
	*Medication or information-related*	■ Avoiding perceived or known side effects of ART use
		■ Lack of information and counseling
**Anticipated barriers to adhering to ART**	*Stigma-related*	■ Anticipated logistical or health systems barriers and related disclosure issues
	*Medication or information-related*	■ Characteristics of current ART formulations
		■ Perceived requirement of a special diet

### Facilitators of ART initiation

The participants identified three main reasons for initiating ART: 1) to live a longer, healthier life; 2) to prevent HIV transmission to a partner and child; and 3) to look “normal/healthy” in order to avoid disclosure and HIV-related stigma.

#### Longer, healthier life

For a large majority, initiating ART represented the ability to live a longer and healthier life. Male participants frequently stressed their improved health so as to continue to provide for their families while female participants frequently contextualized their improved health enabling them to able to care for their children longer and preventing their children from facing orphan-hood.

“The ARVs (antiretrovirals) would reduce the viral load and protect me from opportunistic diseases. I use them to prolong my life and live longer.” (HIV-infected male, 37 years, initiated ART, partner on PrEP)

“It will be sad if I die at this moment since they [his children] may suffer so much. At least, for now there is something that can prolong my life enabling me to see them off to the next level (of education, secondary school)…that is why am grateful and praise ARVs.” (HIV-infected male, 40 years, initiated ART, partner on PrEP)

“My young children also motivated me to start using the drugs, because that was the only way I could live and have the opportunity to take care of them and see them grow up.” (HIV-infected female, 35 years, initiated ART, partner on PrEP)

#### Prevent HIV transmission to partners or children and continue their relationships

Several participants identified their motivation to use ART to prevent HIV transmission to their partners or children, with prevention to children being stressed more commonly by female participants. Furthermore, both male and female participants noted that the use of ART facilitated remaining in their discordant partnerships and allowing their partnerships to thrive.

“The information I got, that even though we are discordant, if I adhere to the ARVs the chances of infecting my partner are lowered, motivated me a lot.” (HIV-infected male, 37 years, initiated ART, partner on PrEP)

“I can now change my mind and add another child now that ARVs, if used effectively, can protect your child.” (HIV-uninfected female, 28 years, initiated PrEP, partner on ART)

"It (using ART) makes me very happy and my husband will also be free with me since I know he will not be infected. It (ART) is beneficial because it can create a good relationship between me and (my) partner." (HIV-infected female, 34 years, initiated ART, partner on PrEP)

#### Appear normal or healthy in order to avoid disclosure, stigma, and discrimination

Many participants, both male and female, wanted to initiate ART in order to look “normal” or “healthy”. Participants noted being perceived as “normal”-looking was important to them, in order to avoid disclosure of their HIV status to their partners or community members. They particularly felt that ART use avoids disclosure of their HIV status thereby improving their interaction with family and community members and preventing social isolation.

"It’s good to take the drugs and that those who take these drugs appear the same like those who are not infected with HIV…People who take ARVs tend to be healthy and thus you cannot easily differentiate them from those who are not HIV infected." (HIV-infected female, 30 years, initiated ART, partner on PrEP)

“[Taking ART] enables you to function and interact well in the community without fear of discrimination or stigmatization; it makes the signs and the symptoms disappear, making it difficult for other people to know your HIV status.” (HIV-uninfected male, 36 years, declined PrEP, partner on ART)

### Barriers to ART initiation

The reasons for declining ART can be broadly classified into: 1) barriers to initiating, and 2) anticipated barriers to adhering to ART.

#### Barriers to initiating ART

The participants identified several inter-related barriers to initiating ART, such as perceived opposition from the community members to widespread ART use, which makes identifying HIV-infected individuals by symptoms difficult, denial about HIV diagnosis, stigma and fear associated with disclosure of HIV status, misconceptions with ART use, and lack of information and counseling.

**Perceived opposition from the community or religious groups:** Interestingly, several male and female participants noted they perceived opposition from some members of their community to widespread ART use. The participants noted that because community members want an easy way of identifying who amongst them is HIV-positive, that they often don’t want infected persons initiating ART because they now appear healthier or “normal.” In other words, “normalization” of appearance with ART use makes it more difficult to know persons who are infected. While it was unclear how strong of an influence such perceived community opinions had on the individuals who had declined initiation of ART, this phenomenon was largely raised as a hypothetical barrier by those who had initiated ART.

“I have heard people complaining that these drugs have made it difficult to know who is positive and who is not. Before they were introduced, the HIV/AIDS symptoms alone could have aroused some suspicions. These drugs somehow cover these symptoms making it difficult to know who is HIV positive and who is not. There is also the fear that such patients who have benefited from these drugs can easily infect others.” (HIV-infected female, 32 years, initiated ART, partner declined PrEP)

Aside from general perceived opposition to ART initiation from community members, some participants, mostly female, specifically stated that some religious groups or belief systems discouraged the use of ART. These groups believe that HIV is the result of witchcraft or wrongdoing, and the groups’ leaders or believers deter HIV-infected individuals from initiating ART. Instead, they advise the HIV-infected individuals to try alternative medicines or rely on god’s mercy to cure them.

“[My mum] associates [serodiscordancy] with satanic forces because she can’t understand the concept of discordant couples and the causes. She believes HIV is a disease caused by Satan.” (HIV-infected female, 34 years, initiated ART, partner on PrEP)

“I was informed by someone that some of them (HIV-infected individuals) get discouraged by some churches or religions that believe that god can save and cure someone from this HIV. So some of them (HIV-infected individuals) don’t see any reason as to why they should take the drugs when an easier option (such as praying) is available.” (HIV-infected female, 35 years, initiated ART, partner on PrEP)

“There are those who also strongly believe in the religious beliefs. There are some religions that do not allow their followers to use western medication. Thus, some of them will decline using the pills because they do not want to go against their religious beliefs…it is all about faith. They believe that if you have faith in God then you should not use the medicines because it is God who heals the sick.” (HIV-uninfected female, 45 years, declined PrEP, partner on ART)

“Some religions and churches discourage against taking the drugs. Such churches may advocate for prayers as the only treatment one should seek…Then there are those who prefer herbal medication to the western medicine.” (HIV-uninfected female, 36 years, initiated PrEP, partner on ART)

**Denial about HIV positive diagnosis:** The majority of male and female participants who initiated or declined ART felt that one of the primary reasons for declining ART initiation was due to denial about the HIV diagnosis. Participants also felt that social stigma surrounding HIV, in turn, fuels this denial.

“When some people have realized that they are HIV-infected, they don’t want to start using these [ART] drugs because they don’t want to accept that they are infected. Sometimes they say that if they visit the hospital, the will be seen by other people who know them.” (HIV-infected female, 23 years, declined ART, partner on PrEP)

“Sometimes a person may believe that s/he is not HIV infected saying that the test kits used to test him/her was not accurate, hence they don’t take the drugs (ARVs) immediately.” (HIV-infected male, 49 years, initiated ART, partner on PrEP)

**Stigma and fear of disclosure of HIV status:** The majority of male and female participants who initiated or declined ART felt that stigma associated with an HIV diagnosis, as well as stigma associated with taking ART, inhibits the HIV-infected individuals in sharing their diagnosis with their partners, families, and community members, and prevents them from starting ART. Fears of discrimination, marginalization, abandonment, and physical harm prevented persons from disclosing their HIV status or their use of ART. Many of the participants explained how ART use inadvertently discloses one’s HIV positive status, particularly to their partner, as they felt it would be too difficult to take the drugs clandestinely. Being reluctant to disclose their status or ART use to others meant delaying or declining ART initiation.

“People talk, they call those who are HIV positive ‘Jaandilo’ (a DhoLuo word meaning those who swallow or take ARVs)…They say those taking ARVs are useless, have no future, and are hopeless…positive people should stay alone in isolation. People don’t think of them as normal human beings.” (HIV-infected male, 40 years, initiated ART, partner on PrEP)"There is no way you can take the drugs daily without your partner noticing. So they choose not to take the drugs." (HIV-infected female, 40 years, declined ART, partner declined PrEP)

“Some would want their status to remain a secret and risk blowing it all up incase their partners find these drugs (ARVs). So they would rather not take the pills. Some find it difficult to inform their partners of their HIV status fearing what their reactions will be. It is easier to inform your mother than the husband.” (HIV-infected female, 35 years, initiated ART, partner on PrEP)

“At first it was a great challenge [in initiating ART] for I feared meeting other people who know me [at the health center], such as friends, relatives, and colleagues, but don’t know my HIV status…I feared that they would disclose my HIV status back at home and leave me subjected to ridicule.” (HIV-infected female, 22 years, initiated ART, partner declined PrEP)

"I will not feel comfortable walking around the community if people have a poor image of me…if taking the pills might make people know your status, then it would be better if you do not take them, so that you just die. That’s the reason why some people might refuse to take the pills." (HIV-infected male, 46 years, declined ART, partner declined PrEP)

“The appearance of these drugs (in a homestead) is truly a scare for most people…People, especially those who are not well informed about the drugs, may even avoid visiting the home, fearing that they may get infected.” (HIV-infected female, 35 years, initiated ART, partner on PrEP)

**Avoiding perceived or known side effects of ART use:** Many male and female participants who initiated or declined ART identified avoidance of perceived or known side effects of the ARVs as another major barrier to ART initiation. These perceived side effects were largely physical in nature, such as rashes, headaches, nausea, etc.

“Some people say it (ART) can make a person to have nausea most of the time. For instance, most women who use the drugs vomit frequently. Sometimes it can also make you to appear so fat but indeed you are not.” (HIV-infected female, 27 years, initiated ART, partner on PrEP)

"Some people also say that when you start using the drugs (ART) you can experience rashes all over your body. I would not like to have the rashes on my body. I have chosen on rashes because they will make me look dirty and I will lose my self-esteem and would not want to even mingle with people around me. At least in the case of swollen legs I can give an excuse that something hit my leg but for the rashes people will definitely start questioning if am HIV positive." (HIV-infected female, 40 years, declined ART, partner declined PrEP)

Ironically, weight gain was often perceived as a negative side effect, largely because of the “Lazarus effect” with ART initiation—previously wasting and emaciated individuals once they initiate ART gain weight back. Such weight gain, subsequently, may signal to their families and community members that they were indeed HIV-infected; many perceived such inadvertent disclosure as undesirable.

“There are some [ARVs] which causes skin rashes in a patient’s body. I have also witnessed a certain lady who has gained so much weight since she started the ARVs. As much as this is a benefit, it is also a disadvantage as it exposes a patient who is on ARVs to the public and he/she can be suspected. When people see an individual who was small bodied but suddenly has gained so much weight, to the extent of becoming shapeless, the conclusion is that such an individual is on ARVs.” (HIV-uninfected female, 31 years, initiated PrEP, partner on ART)

**Lack of information and counseling:** While most participants were aware of ART and its benefits, several male and female participants who had initiated ART hypothesized that the lack of information and counseling about ART could explain why other HIV-infected individuals delay initiating ART. However, those who had actually declined ART initiation did not commonly cite this reason.

“Another thing is lack of information. Some people think that HIV/AIDS is no different from death itself. Some people see HIV/AIDS as a death sentence. Some of the health workers at the volunteer counseling and testing centers also don’t provide enough information and counseling to their clients. Such clients suffer a lot when they test positive.” (HIV-infected female, 35 years, initiated ART, partner on PrEP)

#### Anticipated barriers to adherence

In addition to the several barriers identified in initiating ART, some participants also noted several anticipated barriers to continued adherence, which then prevented them from initiating ART in the first place. Participants, both male and female, found it difficult to adhere to ART due to the following barriers: anticipated logistical barriers and related disclosure issues, unpleasant characteristics of the medications, and perceived requirement of a special diet.

**Anticipated logistical or health systems barriers and related disclosure issues:** Many participants anticipated several logistical barriers when trying to adhere to ART. The first set of logistical barriers centered on obtaining the ARVs or their refills. The anticipated expenses for and travel time to, as well as wait times at, the facilities discouraged some participants in initiating treatment. Many also stated that they preferred to travel to a facility further away to seek ART to prevent inadvertent disclosure to their community members, which, in turn, complicated their ability to travel easily to that facility and adhere to ART.

“I think it (delay in receiving drugs at center) is one thing that brings me to procrastinate in starting taking the pills (ARVs). People will see me as I wait for the pills. You will hear people say ‘these people are waiting for pills’. I don’t like that…People will be seeing us as HIV positive individuals. That makes me unhappy.” (HIV-infected male, 46 years, declined ART, partner declined PrEP)

“Sometimes patients wait too long before they are given the pills…If someone comes to the clinic early, then he should also leave early enough. It should not be a case where someone comes to the clinic at 9am and leaves the clinic at 1pm, and at times even without the pills!” (HIV-infected male, 46 years, declined ART, partner declined PrEP)

“(Female) People are scared of being seen at the hospital collecting the (ARV) drugs…They fear that if seen, they will be exposed to gossip in the community…(Male) Some people, because of that fear, resolve to seek medication from far places where they are not known, but as a result may face the challenge of meeting the transportation costs.” (HIV-infected female, 33 years, initiated ART; HIV-uninfected male, 37 years, initiated PrEP)

The second set of logistical barriers centered on disclosure while seen taking the ARVs outside of the home, including at work. Some participants highlighted a fear of inadvertently disclosing their HIV-status to their co-workers and therefore preferred avoiding taking the pill at work. For some persons this was very difficult, as their work required them to travel for days at a time.

“It also becomes a challenge to take the pills in meeting places. For instance, when you are in attendance in a burial ceremony, it won’t be easy to have the medication in the public. It raises suspicion when you are seen taking a given medication regularly at a particular time.” (HIV-uninfected female, 31 years, initiated PrEP, partner on ART)

“At times, the time for taking the pills might coincide with your daily activities. You might not be in the house as expected. It may prove as a challenge when you are travelling, for instance, and you lack water or maybe the patient fears being seen taking the pills.” (HIV-uninfected male, 24 years, initiated PrEP, partner declined ART)

“Maybe the person might have travelled elsewhere and does not want people to know that s/he is taking these drugs. Even if the person was taking the drugs daily, then it forces him/her to skip that day. That’s why they take it in bits as they skip.” (HIV-infected female, 22 years, declined ART, partner on PrEP)

**Unfavorable characteristics of current ART formulations:** A majority of the participants found it cumbersome to take one or more pills on a daily basis and for the long-term.

“(Male) What people fear is taking the medication for life. (Female) And on a daily basis.” (HIV-infected male, 37 years, initiated ART; HIV-uninfected female, 31 years, initiated PrEP)

"Some people also don’t like the fact that they will have to take the pills on daily basis that when they wake up that is the first thing to come across. So it’s a difficult to start taking the drugs because you will have to keep thinking of taking these drugs most of the time." (HIV-infected female, 27 years, initiated ART, partner on PrEP)

Many participants, particularly those who had initiated ART but also others who had declined ART initiation, found the size, color, or odor of the pill to be particularly bothersome. For example, a women who had declined ART initiation noted hearing several negative characteristics, such as the ARVs being too large and difficult to swallow, from those she knew who were taking ART. She further elucidated odor as the most bothersome characteristic for her:

“Its (ARVs’) smell is bad and the smell does not get over quickly. To me, the big size is not a major problem, because once you have swallowed it you can’t see or feel it, but you can still feel the bad smell in you.” (HIV-infected female, 19 years, declined ART, partner declined PrEP)

**Perceived requirement of a special diet:** Some participants who had initiated ART stated that they were counseled to consume a “special” diet with their ARVs. This requirement for a special diet, in turn, created a sense of food insecurity, and some participants felt that maintaining such dietary requirements was unsustainable. Therefore, they stated that the sense of food requirements and insecurity may act as hypothetical deterrents to ART use for those declining ART.

“There is also the issue that these ARV drugs need good diet. Some people’s incomes are so low that they cannot afford to maintain the diet as required. To them this whole experience with the drugs will be expensive to maintain therefore they would rather not take them at all.” (HIV-infected female, 35 years, initiated ART, partner on PrEP)

## Discussion

This study identified several facilitators and barriers to ART use among heterosexual discordant couples in Kisumu. We identified three key facilitators to ART initiation and adherence: 1) living a healthier life; 2) preventing transmission to partners and/or children; and 3) appearing “normal” or “healthy” again. However, this study also identified two leading sets of barriers to ART initiation or adherence. First, participants noted HIV-related stigma and disclosure issues deterred ART use and adherence, including perceived community opposition to ART use. Second, characteristics of the ARVs, their perceived side effects, and logistical/health systems barriers in obtaining and inadvertent disclosure in taking ART publicly prevented others from initiating and adhering to ART.

The most salient finding in our study is how pervasively HIV-related stigma continues to influence HIV-positive individuals, including in their ART initiation decision-making. Disclosure of HIV-positive status and potential consequences of associated stigma act as significant barriers to ART initiation. Furthermore, ART use, due to the physical act of taking oral pills on a daily basis, allows HIV-infected individuals to be identified, inadvertently disclosing their positive status—a phenomenon that both participants who initiated and declined ART raised as a major barrier in ART initiation. Other studies have noted similar findings, identifying stigma associated with taking ART, due to inadvertent disclosure, as a significant barrier to ART initiation [21–28]. While the global community has made great strides in reducing HIV-related stigma and discrimination, our study is a sober reminder that greater efforts need to be taken to further reduce stigma so that inadvertent disclosure of HIV status does not take such prime importance in individuals’ decision-making to initiate ART.

Another interesting finding from our study is that appearing “normal” or “healthy” with ART use was considered a double-edge sword, due to its interrelatedness to disclosure and stigma. Losing weight, for example, due to HIV-related wasting in an infected individual not on ART, may hint to others this person’s HIV status. On the other hand, gaining weight after ART initiation may also signal to others the individual’s HIV status. Participants acknowledged that becoming “normal” or “healthy” again was an important motivation to start ART, as other studies have shown[21, 22, 25]. However, we report a new finding that the change in their physical health and appearance might inadvertently disclose their HIV status, which negatively impacts ART initiation or adherence. As individuals initiate ART early at higher CD4 counts or health status, this type of “Lazarus effect,” of being deathly ill, loosing weight, and then regaining weight with ART use, should become less common.

We also found that many of the participants perceived community opposition in starting ART for several reasons. First, because ART facilitates HIV-infected individuals to appear “normal” or “healthy” again, these individuals may no longer be easily identified by their community members as being “ill” with HIV. Therefore, infected individuals perceive some community members as being opposed to their starting ART. It appears that some religious leaders or sects may dissuade HIV-infected individuals from initiating ART because they associate HIV and/or ART use with witchcraft or going against God’s will[21, 22, 24, 28]. Greater sensitization at the community level, including with religious leaders, is needed to remove misconceptions and avoid perpetuating a culture of fear and stigma for HIV-infected individuals.

Additional barriers to ART initiation were anticipated barriers to ART adherence. Our results concur with others that daily and long term pill use can be burdensome to many HIV-infected individuals [21, 22, 24, 28]. The pill characteristics, such as size, odor, or color, also caused discomfort for some participants [24, 26, 29]. In addition, the potential need to follow a special diet while taking ART prevented others from starting ART. The perceived side effects of ART use prevented some from initiating ART [17, 22, 24–26]. Here too, participants were concerned about inadvertent disclosure of their positive status if others saw them experiencing commonly known side-effects of ART [21, 24]. Lastly, logistical or health systems issues such as the need to and expense of travel to facilities to obtain refills posed a barrier to ART adherence [2, 22, 24, 27, 28, 30, 31]. Increasing visit or refill durations, improving pill characteristics, and longer-acting formulations can reduce anticipated barriers to ART adherence and facilitate ART initiation.

We also found several factors that facilitated ART initiation. The benefits of ART, in terms of improving health status, life span, and quality of life and preventing transmission to infants or partners, motivated many participants to initiate ART. Interestingly, conceptualization of the consequences of improved health occurred within gender labor norms of the male as “breadwinner” and the female as “care taker” of their families, as delineated in Connell’s Theory of Gender and Power [32]. HIV treatment programs can build messaging and counseling strategies around these positive themes to help motivate HIV-infected individuals to initiate ART.

Ultimately what our findings reveal is the interconnectedness of individuals to their social networks and contexts is key in influencing ART initiation decision-making, and how normative beliefs strongly influence personal beliefs. A prominent finding in our study is how commonly participants responded to questions by discussing what they thought others were thinking; perceived normative beliefs greatly influenced their thinking. Some theoretical frameworks employed in understanding ART initiation and adherence, such as the Theory of Planned Behavior, have stressed the importance of social norms in influencing behavior intention and the subsequent behavior [33]. However, what our findings also reveal is an even more complex interconnectedness between these individuals and their social networks, that goes beyond social norms influencing personal beliefs. As such, we suggest that theoretical frameworks that capture the social fabric of these individuals better, such as the Individual-Family-Community model [34, 35], which posits that individual behavior, specifically ART adherence, is best supported by resilient families and competent communities, should be further explored to better understand decision-making around ART initiation and adherence instead of focusing on individual-level only influences.

There are several strengths of this study. First, we conducted the interviews with participants who were eligible for ART initiation, many of whom subsequently declined ART initiation, rather than querying hypothetical ART use. Second, we conducted the study in a high HIV prevalence setting among heterosexual discordant couples, which allowed us to elicit aspects of relationships that influence ART initiation decision-making. Third, we conducted this study with a relatively large sample size, which ensured adequate saturation of themes. We sampled participants from only one geographic region of Kenya, however, which limits the generalizability of the results. Our results require validation with a larger and more varied sample of providers and patients.

## Conclusions

Among heterosexual discordant couples in Kisumu, Kenya, we found that living a healthier life, preventing HIV transmission to others, and appearing “normal” or “healthy” again motivated infected individuals to initiate ART. We also found that these individuals perceived community opposition to their initiating ART and appearing “normal” or “healthy” again, because, ironically, community members would not be able to discreetly identify infected individuals in their community anymore. Such perceived stigma and issues with disclosure continue to hinder ART initiation among infected individuals. Community sensitization strategies need to continue to address fear of disclosure and stigma that infected individuals perceive. In addition, infected individuals anticipate several barriers to adhering to ART, such as side-effects or challenges in obtaining refills, which, in turn, prevent these individuals from initiating ART. As programs in resource-limited settings develop plans to scale-up ART use for all HIV-infected individuals, addressing these barriers to ART initiation and adherence should be central to their efforts.
